# Association of TGF-β Canonical Signaling-Related Core Genes With Aortic Aneurysms and Aortic Dissections

**DOI:** 10.3389/fphar.2022.888563

**Published:** 2022-04-20

**Authors:** Jicheng Chen, Rong Chang

**Affiliations:** Department of Vasculocardiology, Shenzhen Longhua District Central Hospital, Guangdong Medical University, Shenzhen, China

**Keywords:** TGF-β signaling, aortic aneurysm, aortic dissection, SMADs, smooth muscle cells

## Abstract

Transforming growth factor-beta (TGF-β) signaling is essential for the maintenance of the normal structure and function of the aorta. It includes SMAD-dependent canonical pathways and noncanonical signaling pathways. Accumulated genetic evidence has shown that TGF-β canonical signaling-related genes have key roles in aortic aneurysms (AAs) and aortic dissections and many gene mutations have been identified in patients, such as those for transforming growth factor-beta receptor one TGFBR1, TGFBR2, SMAD2, SMAD3, SMAD4, and SMAD6. Aortic specimens from patients with these mutations often show paradoxically enhanced TGF-β signaling. Some hypotheses have been proposed and new AA models in mice have been constructed to reveal new mechanisms, but the role of TGF-β signaling in AAs is controversial. In this review, we focus mainly on the role of canonical signaling-related core genes in diseases of the aorta, as well as recent advances in gene-mutation detection, animal models, and *in vitro* studies.

## Introduction

Aortic aneurysm (AAs) and aortic dissections (ADs) are degenerative vascular diseases associated with high mortality. The main pathological manifestations are degeneration of the media layer, extracellular matrix (ECM) remodeling, infiltration of inflammatory cells into the aorta, progressive dilation of the aortic diameter and, finally, sudden death of the patient due to aorta rupture (Rabkin, 2017; Bossone and Eagle, 2021). AAs and ADs account for 1–2% of all deaths in Western countries, aortic aneurysm is the second most prevalent aortic disease following atherosclerosis and accounts for the ninth-leading cause of death overall, the estimated incidence is 2.79 per 100 000 individuals (Sampson et al., 2014).

In recent years, accumulating evidence has indicated that genetic factors, particularly transforming growth factor (TGF-β) signaling, play a key role in the development of aortic aneurysms, in addition to regulating many aspects of physiological homeostasis in embryonic and adult tissues (Lindsay and Dietz, 2014; Isselbacher et al., 2016; Tzavlaki and Moustakas, 2020). Several mutated genes have been identified from patients with AAs, many of these mutated genes belong to members of the TGF-β signaling family. However, the role of TGF-β signaling in AAs is controversial (Jones et al., 2009; Lin and Yang, 2010).

In this review, we focus mainly on the role of canonical signaling-related core genes in diseases of the aorta.

## Overview of TGF-β Signaling

TGF-β superfamily is one of main signaling pathways in humans. Initially, it was found to have a crucial role in growth and development. Subsequent studies revealed it to be involved in regulation of various cellular processes: proliferation, differentiation, adhesion, metastasis, and apoptosis (Wu et al., 2014; Meng et al., 2016; Tzavlaki and Moustakas, 2020). In recent years, it has been revealed that dysregulation of the TGF-β signaling pathway is closely related to several human diseases.

The transforming growth factor-beta (TGF-β) superfamily is comprised of over forty members, such as TGF-βs, anti-mullerian hormone, nodal, activin, and bone morphogenetic proteins (BMPs), growth differentiation factors (GDFs). It can be divided into two subfamilies: TGF-β/Activin/Nodal and BMP/GDF/MIS (Muellerian inhibiting substance) (MacFarlane et al., 2017; Miyazawa and Miyazono, 2017).

There are three types of TGF-β ligands, TGF-β1, TGF-β2 and TGF-β3, and they have amino-acid homology of 64–82% (Perrella et al., 1998). Expression of these ligands is specific to tissues. TGF-βs undertake very important regulatory process in the ECM. If an inactive cleaved peptide fragment, such as latent associated protein (LAP), is secreted by cells, it forms a large latent complex (LLC) with latent transforming growth factor binding protein (LTBP), Fibrillin-1 binds LTBP and anchors LLC to the ECM (Wrana et al., 1992; Isogai et al., 2003; Shi et al., 2011). TGF-βs can activate only the corresponding receptor after dissociation from LAP; this regulatory process can concentrate and regulate the concentration of TGF-βs to meet the different needs of cells.

TGFΒR1 and TGFΒR2 are single transmembrane glycoproteins. Their cytoplasmic regions have serine/threonine kinase activities. Seven type I receptors and five type II receptors have been identified in humans (Heldin and Moustakas, 2016). Binding of TGF-βs, activin or nodal to receptors can lead to activation of canonical signals and TGFΒR2 can trans-phosphorylate and activate TGFΒR1 to form activated receptor dimers. Downstream SMADs proteins are activated further by activated receptor dimers (Wrana et al., 1992; Peng, 2003). The SMADs protein family is present in the cytoplasm and can transmit signals from the cell membrane directly to the nucleus. They can be divided into three subfamilies according to their structure and function: (i) receptor-activated SMADs (R-SMADs), including SMADl, 2, 3, 5, 8, and 9; (ii) common-partner SMADs (Co-Smads), only SMAD4 in mammals can intact with activated R-SMADs, and is the core transcription factor of the TGF-β signal; (iii) inhibitor SMAD (I-SMADs), including SMAD6 and 7, which block receptor-mediated phosphorylation of R-SMAD and inhibit the formation of activated R-SMAD and Co-SMAD isopolymers. SMAD6 preferentially inhibits SMAD signaling initiated by the bone morphogenetic protein (BMP) type I receptors ALK-3 and ALK-6, whereas SMAD7 inhibits both transforming growth factor β (TGF-β)- and BMP-induced Smad signaling (Hanyu et al., 2001; Goto et al., 2007; Miyazawa and Miyazono, 2017). I-SMADs can also bind competitively to activated receptors or R-SMADS to form inactive complexes, thereby inhibiting Co-SMAD-mediated gene expression (Derynck and Zhang, 2003; Park, 2005; Hata and Chen, 2016).

In the inactive state, SMAD4 is distributed in the cytoplasm and nucleus, R-SMADs are located mainly in the cytoplasm, and I-SMADs are mainly in the nucleus. If the receptor is activated, SMAD2 and SMAD3 are phosphorylated and activated to form a complex and transferred to the nucleus. The phosphorylated SMAD2/3 interacts with SMAD4 to form a Smad complex and synergistically regulates transcription of the target gene with other transcription factors (Moustakas et al., 2001; Feng and Derynck, 2005).

**FIGURE 1:**
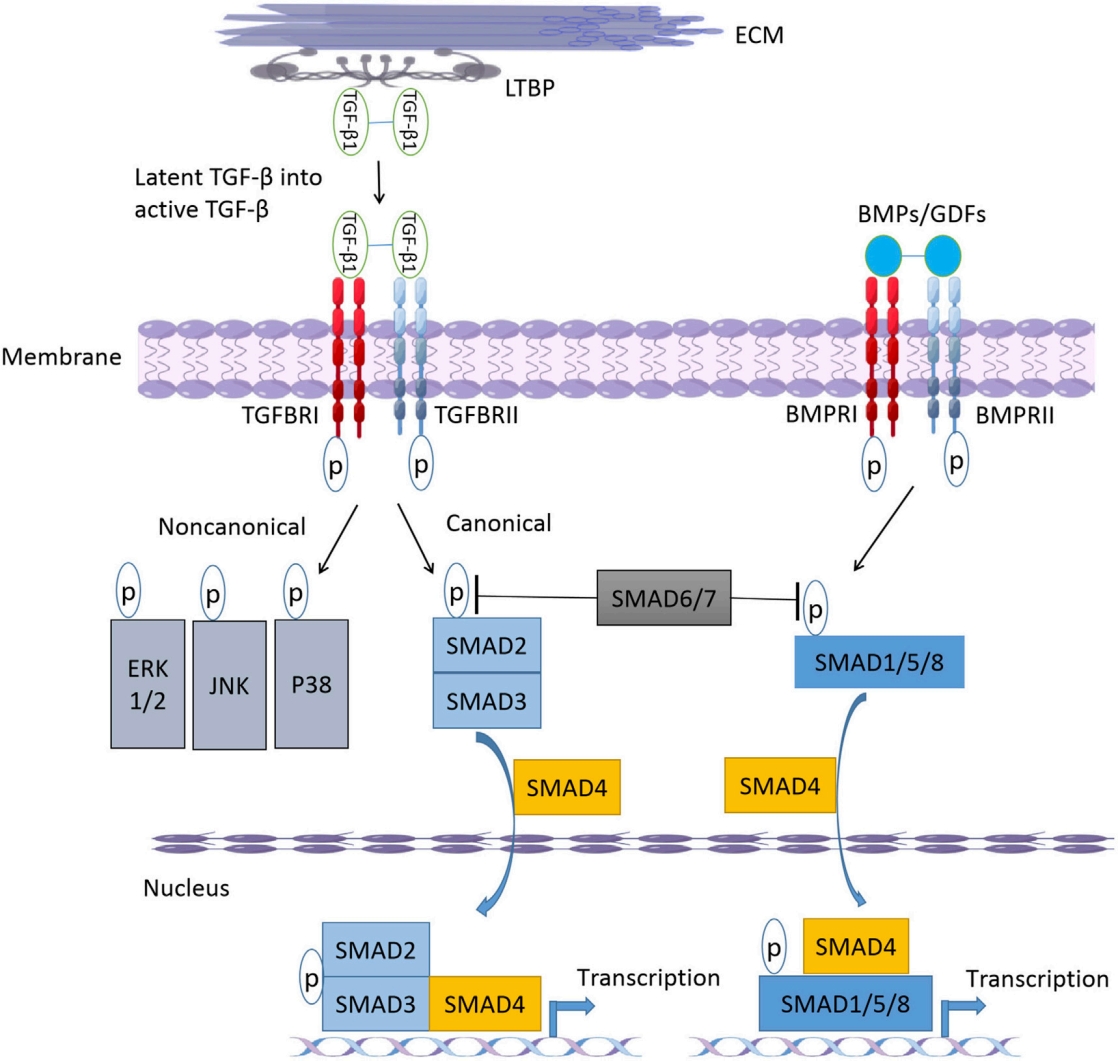
The-β/BMP signaling pathway. After cellular secretion, latent TGF-β forms a large latent complex (LLC) with latent transforming growth factor binding protein (LTBP). Upon release from the LTBP, TGF-β binds to receptors and TGFBR2 can trans-phosphorylate and activate TGFBR1 to form activated receptor dimers. Activation of receptor complex initiates canonical and non-canonical TGF-β signaling pathways. After binding to active ligands BMPs or GDFs, BMPR1 and BMPR2 form phosphorylated dimers and activate downstream SMAD1/5/8. SMAD6 and SMAD7 can inhibit TGF- β and BMP signaling pathways by inhibiting SMAD2/3 and SMAD1/5/8 phosphorylation, respectively. ECM, extracellular matrix; TGF- βs, transforming growth facotr-beta; TGFBRs, transforming growth factor-beta receptors; BMPs, Bone morphogenetic proteins; GDFs, growth and differentiation factors; SMADs, *drosophila* mothers against decapentaplegic proteins; ERK, extracellular signal-regulated kinases; JNK, c-Jun N-terminal kinase.

The downstream signal transduction of BMP differs from TGF-β signal in that it depends on SMAD1/5/8. BMPs and GDFs bind to BMP type I receptors, including ALK-1 (activin receptor-like kinase 1), ALK-2, ALK-3/BMPRIA, or ALK-6/BMPRIB, nodal signals through ALK-4, the major type I receptor for activins, whereas myostatin signals through ALK-4 or ALK-5 (Table 1) (Morikawa et al., 2016), and then activate SMAD1/5/8 to form a complex with SMAD4 (Figure 1). The Smads complex translocates to the nucleus and regulates BMP-specific target genes, while inhibitory ligands, such as Noggin, block ligand-receptor binding and pathway activation (Chang et al., 2002). However, the pairing between receptors and ligands is not absolute, but there is some crossover. For example, TGF-βs-activated TGFΒR2 not only interacts with TGFΒR1, but also binds to ALK-1, thus leading to activation of both SMAD2 and SMAD3, also SMAD1, SMAD5, and SMAD8 in cells that express both ALK-1 (Goumans et al., 2003).

**TABLE 1:** Protein-receptor pairing in the TGF-β superfamily.

Ligands	Type I receptors	Type II receptors
TGF-βs	TGFBRI (Alk5)	TGFBRII
Nodal	AcvR-lb, AcvR-IC	AcvR-II, AcvR-IIb
Myostatin	AcvR-lb, TGFBRI (Alk5)	AcvR-II, AcvR-IIb
Activin	TGFBRI (Alk5)	AcvR-II, AcvR-IIb
BMPs, GDFs	AcvRL-I (ALK1), AcvR-I (Alk2)	AcvR-II, AcvR-IIb, BMPR-II
	BMPR-la (Alk3)	—
	BMPR-lb (Alk6)	—

TGFBRs, transforming growth factor-beta receptors; AcvRs, activin A receptors; BMPs, Bone morphogenetic proteins; GDFs, growth and differentiation factors; AcvRL-I, activin A receptor like type 1; BMPRs, bone morphogenetic protein receptors.

Studies have shown that SMADs in the TGF-β canonical signaling pathway play a very important part in AAs and ADs, and many gene mutations have been identified in patients (Takeda et al., 2018). Studies in animal models have revealed some new molecular mechanisms. In this review, we focus on the research progress of these core genes in AAs and ADs. In addition, TGF-β can activate many other signaling pathways that do not involve SMADs directly: extracellular-signal regulated kinase 1 and 2 (ERK1/2), c-Jun N-terminal kinase (JNK) (Lee et al., 2007; Sorrentino et al., 2008; Yamashita et al., 2008), phosphoinositide 3-kinase-Akt (PI3K-Akt) (Wilkes et al., 2005), and p38 mitogen-activated protein kinase (MAPK) (Li and Kong, 2020). We do not elaborate on TGF-β non-classical signaling pathways.

## TGFΒR1 and TGFΒR2 Genes

TGFΒR1 is located on chromosome 9q. TGF**Β**R2 is located on chromosome 3p. TGFΒR1 and TGFΒR2 are transmembrane proteins with serine/threonine kinase motifs, containing nine and seven exons, respectively. Early sequencing studies showed pathogenic mutations of TGFΒR1 and TGFΒR2 genes to be associated with a series of other pathological changes in addition to AAs, named Loeys–Dietz syndrome 1 (LDS1) and LDS2, respectively. Table 2 gives an overview of important genetic defects leading to AAs and ADs formation in human. Most mutations in TGFΒR1 and TGFΒR2 genes are loss-of-function mutations, mainly in the evolutionarily conserved serine/threonine kinase region. One-third of the TGFΒR mutations are identified in TGFΒR1, whereas the remainder is found in TGFΒR2 (Loeys et al., 2005; Pannu et al., 2005; Loeys et al., 2006).

**TABLE 2:** Loss-of-function genetic defects causing AAs and ADs in human.

Gene	Chromosomal locus	Associated syndrome	Main clinical features	References (Pmid)
TGFBR1	9q22.33	Loeys-Dietz syndrome type I	Widespread arterial aneurysms with tortuosity ND ortic dissections occur earlier, pectus deformities, scoliosis, arachnodactly, craniosynostosis, hypertelorism, cleft palate or bifid uvula, club feet	15731757
				16928994
TGFBR2	3p24.1	Loeys-Dietz syndrome type II	Wildespread arterial aneurysms with tortuosity and aortic dissections occur earlier, pectus deformities, scoliosis, arachnodactyly, craniosynostosis, hypertelorism, cleft palate or bifid uvula, club feet	15731757
				16928994
SMAD2	18q21.1	Loeys-Dietz syndrome type IV	Arterial aneurysms and dissections, valve abnormalities, hypertelorism, scoliosis, complex cogential heart disease	26247899
SMAD3	15q22.33	Aneurysm-osteoarthritis syndrome	Wildspread and aggressive arterial aneurysms and dissections, arterial tortuosity, early-onsetosteoarthritis, hypertelorism, bifid uvula	21217753
				22167769
SMAD4	18q21.2	Juvenile polyposis syndrome and hereditary hemorrhagic telangiectasia	Aortic dilatation, Gastrointestinal hamartomatous polyps, epistaxis, telangiectasia and arteriovenous malformations	28874282
SMAD6	15q22.31	Bicuspid aortic valve	Thoracic aortic aneurysm and dissections, bicuspid aortic valve, aortic isthmus stenosis, dilated cardiomypathy, hyperplasia of cardiac valves	28659821
				32748548
SMAD7	38.p13	No report	—	—

AAs, Aortic aneurysms; ADs, Aortic dissections; TGFBRs, transforming growth factor-beta receptors; SMDA, drophila mothers against decapentaplegic proteins.

LDS is an autosomal-dominant genetic disorder. Although LDS shows clinical overlap with Marfan syndrome (MFS), it can be distinguished clinically from MFS (Meester et al., 2017). Except for AAs, the common features include pectus deformities, scoliosis, and arachnodactyly. Distinguishing findings are craniosynostosis, hypertelorism, cleft palate or bifid uvula, club feet, instability in the cervical spine and, most importantly, widespread arterial aneurysms with tortuosity and aortic rupture occur earlier. Moreover, in more severe cases of LDS, cervical instability or cleft lip and palate may be present. The arteriopathy observed in LDS is disseminated, with AAs and ADs occurring in peripheral arterial beds and the aorta. Conversely, ectopia lentis (a finding highly specific to dysfunction of fibrillin-1 protein) is common in MFS but is not observed in LDS (Tran-Fadulu et al., 2009; Van Laer et al., 2014; MacFarlane et al., 2017). Interestingly, heterozygous deletion of TGFΒR1 does not cause vascular disease. Heterozygous mutations in the TGFΒR1 gene have been shown to cause multiple self-healing squamous epithelioma (MSSE). Most mutations in MSSE are extracellular ligand-binding domains or truncation mutations of the kinase domain, whereas most mutations in LDS are missense (Goudie et al., 2011; Fujiwara et al., 2019).

Abnormally enhanced TGF-β signaling has been observed in aortic-tissue samples from patients with LDS. Co-transfection of wild-type and mutant TGFΒR1 or TGFΒR2 receptors in equal quantities results in reduced TGF-β signaling, indicating that mutant receptors cannot propagate a signal even in the presence of their wild-type counterpart (Mizuguchi et al., 2004; Horbelt et al., 2010; Gallo et al., 2014). It has been revealed that mice carrying TGFΒR1 and TGFΒR2 heterozygous missense mutations, but not which are not haplo-sufficient for TGF-β receptor alleles, recapitulate LDS phenotypically, which suggests that the presence of mutant TGFΒR is necessary to cause disease (Gallo et al., 2014). Table 3 gives an overview of mouse models associated with disturbed TGF-βsignaling pathway.

**TABLE 3:** Mouse models associated with disturbed TGF-β signaling pathway.

Gene	Mouse model	Phenotype	Mechanism	References (Pmid)
TGFBR1	KO	Embryonic lethal, angiogeneisis defects	—	11285230
	Tgfbr1^M318R/+^ heterozygous missense	Enlarged aortas and accelerated aortic root, recaptitulate LDS	Increased TGF-β signaling contributes to postnatal aneurysm progression, losartan prevents aortic aneurysm	24355923
	Postnatal SMC-specific tamoxifen injection	Aortic rupture and aneurysmal degenration, 100% penetrance of ascending thoracic aortas	Inhibition of ERK phosphorylation or blockade of the ATR1 can prevent aneurysmal degeneration of Tgfbr1-deficient aortas	27739498
	EC-KO	Embryonically lethal, severe defects in vascular development	—	18029401
TGFBR2	KO	Embryonic lethal, angiogenesis defects	—	8873772
	Postnatal SMC-specific	Significantly lower degree of aneurysmal degeneration than TGFBR1 KO	Treatment with rapamycin restored a quiescent smooth muscle phenotype and prevented dissection	24401272
				26494233
				27739498
	Tgfbr1^M318R/+^ heterozygous missense	Enlarged aortas and accelerated aortic root, recapitulate LDS	Increased TGF-β signaling contributes to postnatal aneurysm progression, losartan prevents aortic aneurysm	24355923
SMAD2	KO	Die before embryonic day 8.5 due to defective elongation of egg cylinders and germ-layer formation	—	9529255
				9689088
	Neural crest cells-KO	Reduced numbers of SMCs in the media, thinner elastic lamina, and reduced vessel-wall thickness of carotid arteries	—	23817199
SMAD3	KO	Progressive dilation of aortic roots and the ascending aorta	Adminstration of anti-GM-CSF monoclonal antibodies to KO mice resulted in significantly less dilation in the aortic root	27688095
				23585475
	Angiotensin II-infused SMAD3 KO	Significant aortic dilatation, medical and adventitial thicking	Adminstration of clodronate-liposomes depleted macrophage, and inhibition of iNOS restored elastin content and alleviated aortic dilation	23782924
	CaCI_2_ induced SMAD3 KO	Abdominal aortic aneurysm	Enhanced staining for phosphorylated-SMAD2 and phosphorylated-ERK	25985281
SMAD4	KO	Pre-angiogenesis lethality	—	17895899
	EC-KO	Died at embryonic day 10.5 due to cardiovascular defects	—	17724086
	Postnatal SMC-specific	Aortic aneurysm and dissection, severe inflammatory cells infiltrate into the aorta	Macrophage infiltration contributes to the progression of aortic aneurysms. Therapeutic targeting of IL-1b improved aortic disease	26699655
				29150241
SMAD6	KO	Embryonic and postnatal lethality with moderate penetrance, hyperplastic thickening of the cardiac valves, skeletal defects		30098998
				10655064
SMAD7	KO	Majority die *in utero* due to cardovasacular defects. impaired cardic functions and severe arrhythmia	—	18952608
	Hepatocyte-specific Smad7-knockout	Liver hepcidin expression and develop an iron deficiency phenotype	Increased phosphorylation of SMAD2/3 in atrioventricular cushion and endocardium	29575577

TGFBRs, transforming growth factor-beta receptors; SMADs, *drosophila* mothers against decapentaplegic proteins; KO, knock out; LDS, Loeys-Dietz syndrome; SMCs, smooth muscle, smooth muscle cells; ATR1, angiotensin II, type 1 receptor; EC-KO, endothelial cell-knock out; GM-CSF, granulocyte-macrophage colony stimulating factor; iNOS, inducible nitric oxide synthase; IL-1b, Interleukin-1, beta.

Smooth muscle cell-specific (SMC) knockout of TGFBR1 or TGFBR2 can lead to AAs, but TGFΒR1 knockout is more destructive to the aorta. Enhanced ERK signaling in the aortic wall before causing structural degeneration has been observed in TGFΒR1 SMC knockout mice. Inhibition of ERK phosphorylation or blockade of the angiotensin II type I receptor can prevent aneurysmal degeneration of Tgfbr1-deficient aortas (Yang et al., 2016). In tamoxifen-induced TGFΒR2 SMC knockout mice, loss of the TGF-β signaling pathway impairs the contractile apparatus of vascular smooth muscle, damages elastin, increases the number of macrophage markers, and increases cell proliferation and matrix accumulation (Li et al., 2014; Hu et al., 2015). Angiotensin II-induced disease in the abdominal aorta is exacerbated by systemic TGF-β blockade, and thoracic aortic disease is exacerbated by SMC-specific loss of TGBR2, which suggest that TGF-β signaling prevents abdominal and thoracic aneurysmal disease by different mechanisms (Angelov et al., 2017). Those mouse experiments demonstrated that, as core molecules of the TGF-β signaling family, TGFΒR1 and TGFΒR2 are essential for maintaining the structural integrity of the aorta after normal embryonic development.

### Receptor-Mediated SMADs (R-SMADs)

SMAD2 and SMAD3 mutations are important predisposing factors for AAs and ADs. They are located on the long arms of chromosomes 18 and 15, and are composed of 11 and 9 exons, respectively (Schepers et al., 2018). SMAD2 and SMAD3 proteins belong to the R-Smad family and are important downstream effectors of the canonical TGF-β signaling pathway.

### -SMAD2 Gene

Pathogenic variants in SMAD2 have been reported in to be associated with AAs and ADs in only a few studies. Whole-exome sequencing was undertaken in 365 patients with AAs or ADs without FBN1, TGFΒR1, TGFΒR2, ACTA2, or MYH11 mutations: three SMAD2 loss-of function variants were identified. They were located in the MH2 region of the SMAD2 (Micha et al., 2015). Then, a novel missense mutation in SMAD2 in a Chinese family with early-onset AAs was identified (Zhang et al., 2017). Studies have shown that two distinct phenotypes are associated with pathogenic variants in SMAD2: (i) complex congenital heart disease with or without laterality defects and other congenital anomalies; (ii) a late-onset vascular phenotype characterized by arterial aneurysms with connective-tissue abnormalities (Granadillo et al., 2018). Recently, studies have identified a SMAD2 nonsense variant and four missense variants, which are all located in the MH2 domain. In addition to AAs, these affected individuals present with a connective-tissue pathological phenotype (Cannaerts et al., 2019). Those studies suggest that screening for SMAD2 mutations is warranted in patients with AAs or ADs.

A striking feature is the reported increase in phosphorylated-SMAD2 expression and TGF-β signaling in the aortic wall of patients with AAs or ADs caused by wide types of genetic mutations (Gomez et al., 2009; Fukuda et al., 2018), such as Fibrillin-1, SMAD3, SMAD4, TGFΒR1, TGFΒR2, TGFβ3, and TGFβ2. This has caused controversy regarding the role of TGF-β signaling in terms of the pathogenic mechanisms leading to AAs. Different hypotheses have arisen to address this issue, but experimental validation is needed. Evidence suggests that one of the mechanisms of overactivation of SMAD2 promoters is associated with excess transcriptional activity (a process associated with epigenetic modification of the SMAD2 promoter). This action is accompanied by switching from repression of SMAD2 expression in a healthy state to p53-dependent activation of SMAD2 by switching from myc-dependent aneurysmal VSMCs in humans (Loinard et al., 2014).

Mice embryos with SMAD2 knockout die before embryonic day-8.5 due to defective elongation of egg cylinders and germ-layer formation. Therefore, the pathological condition of the aorta cannot be detected (Waldrip et al., 1998; Weinstein et al., 1998). SMAD2 is critical for VSMC differentiation from NCCs *in vivo*. Specific knockout of SMAD2 in NCCs leads to defective differentiation of NCCs to VSMCs in aortic-arch arteries during embryonic development. Reduced layers and numbers of VSMCs in the media, thinner elastic lamina, and reduced vessel-wall thickness of carotid arteries has been observed in SMAD2 NCC knockout mice (Xie et al., 2013). SMAD3 is important for the differentiation of SMCs from mesenchymal progenitors (Qiu et al., 2005). Evidence of AAs or ADs caused by SMAD3 deletion has not been reported in mouse models, which may be related to the compensatory effect caused by other genes. In future studies, tissue-specific knockout of SMAD3 and induction with other chemical agents (e.g., angiotensin II or CaCl_2_) may permit discovery of this association in mice.

### -SMAD3 Gene

SMAD3 plays an indispensable part in the homeostasis, remodeling, and fibrosis of connective tissue. The N-terminal MH1 domain of SMAD3 protein mediates binding to DNA, and the C-terminal MH2 domain is involved in protein–protein interactions (Schepers et al., 2018). Mutation of the MH2 domain of SMAD3 was first identified to cause AAs and ADs in patients (van de Laar et al., 2011). In contrast with other aneurysm syndromes, most of these affected individuals presented with early-onset osteoarthritis. Therefore, the syndrome caused by SMAD3 mutations is called “aneurysms–osteoarthritis syndrome” (AOS). Because of the many common clinical features with LDS (hypertelorism, bifid uvula, arterial tortuosity, and widespread AAs and ADs), it is now also classified as “LDS type 3” (van de Laar et al., 2012; MacCarrick et al., 2014). AOS is an autosomal-dominant connective-tissue disorder characterized by AAs and tortuosity, early-onset osteoarthritis, as well as mild craniofacial, skeletal and cutaneous anomalies. However, not all patients with a SMAD3 mutation present with osteoarthritis (Regalado et al., 2011). In contrast to MFS, cerebrovascular abnormalities occur frequently in AOS and LDS (van der Linde et al., 2012). Studies have further reported AOS to be caused by mutations at other SMAD3 sites (Liao et al., 2018).

SMAD3 mutations are responsible for 2% of familial thoracic AAs and ADs (Regalado et al., 2011). Sixty-seven mutations have been identified in SMAD3, including missense, nonsense, frameshift, and splice-site mutations, which are spread over the entire gene. There are no significant “hotspots” for SMAD3 mutations, but most of the reported haploinsufficiency or missense variants are concentrated in the MH2 domain. The latter is responsible for the direct binding of SMAD3 and SMAD4 to form a complex that mediates transcriptional regulation of TGF-β downstream signals (Zhang et al., 2015; Schepers et al., 2018).

A SMAD3 knockout model in mice was used to elucidate the molecular mechanism of SMAD3 involvement in AA pathogenesis. Significant aortic dilatation, medial and adventitial thickening as well as luminal enlargement were observed in angiotensin II-infused SMAD3 knockout mice. Increased vascular inflammation (not hypertension) initiated the AAs. AngII-infused SMAD3 mice showed increased inducible nitric oxide (iNOS)-derived production of nitric oxide and macrophage infiltration in aortas. Administration of clodronate-liposomes depleted macrophage/monocytes, and inhibition of iNOS by aminoguanidine restored elastin content and alleviated aortic dilation significantly (Tan et al., 2013). In another study, SMAD3 deficiency led to progressive dilation of aortic roots and the ascending aorta in adult C57BL/6 mice even without induction by angiotensin II. Smad3^−/−^ (SMAD3 translation-initiation site was deleted) mice had a vascular phenotype similar to that observed in AOS: the aorta had chronic infiltration of inflammatory cells. Granulocyte macrophage-colony stimulating factor (GM-CSF) oversecreted from CD4^+^T cells in Smad3^−/−^ mice has a key role in AA pathogenesis. GM-CSF has been shown to induce monocyte accumulation in the aortic root. In addition, administration of anti–GM-CSF monoclonal antibodies to Smad3^−/−^ mice resulted in significantly less matrix metallopeptidase nine activity and dilation in the aortic root. GM-CSF overexpression has also been detected in the aorta of AOS patients with SMAD3 mutations (Ye et al., 2013). Those data strongly suggest that SMAD3 deletion in peripheral inflammatory cells is an important cause of AA, but we cannot exclude the contribution of SMAD3 deficiency in SMCs to AA occurrence. Therefore, further studies on the pathogenesis of tissue-specific SMAD3 deficiency or mutation must be done.

Another study also supported the role of SMAD3 in protecting vessel-wall integrity and suppressing inflammation in the pathogenesis of CaCl_2_-induced AAAs. CaCl_2_ treatment induced robust infiltration by T cells and macrophages, activation of nuclear factor-kappa B and ERK 1/2 signaling pathways, and upregulation of SMAD 2/4 expression in the abdominal aorta of Smad3^−/−^ mice (Dai et al., 2015). Interestingly, there was no increase in ECM accumulation, excessive collagen accumulation, or loss of SMCs in Smad3^−/−^ mice. The aorta of Smad3-deficient mice showed enhanced staining for phosphorylated-SMAD2 and phosphorylated-Erk, but expression of TGF-β-activated target genes was not upregulated. Instead, the aortas of fibulin-4^R/R^-deficient mice show increased ECM remodeling and a greater downstream transcriptional response (Ye et al., 2013). Those data further suggested that different mutations in patients with AA had different pathogenesis. Switching of the VSMC phenotype also plays an important part in TAAD. miR-21 expression has been found to be increased in an angiotensin II-infused Smad3^+/−^ TAAD mouse model and in patients with ascending TAA. miR-21 deficiency in Smad3^+/−^ mice exacerbates TAAD formation after infusion with angiotensin II; the increased SMAD7 expression and suppressed canonical TGF-β signaling causes SMCs to switch from a contractile phenotype to a synthetic phenotype. Silencing of SMAD7 expression with lentivirus can prevent angiotensin II-induced TAAD formation in Smad3^+/−^ miR-21^−/−^ mice (Huang et al., 2018). Taken together, those independent studies using different lines of SMAD3 knockout mice demonstrated that SMAD3 is critical for protecting vessel walls from aneurysm formation. An excessive inflammatory response is a common feature in AA development, immunosuppression may be an effective option for treatment of patients with SMAD3 mutations.

As the core component of aortic walls, SMC disorders are an important cause of AA formation. Several *in vitro* studies have explored the role of SMAD3 in SMCs. SMAD3 has a vascular-protective role because it regulates vascular SMCs and matrix regulation. c-Ski inhibits the proliferation of aortic smooth muscle in A10 rats by suppressing SMAD3 signaling (Li et al., 2013). Furthermore, miR-26b suppresses AD development by targeting regulation of the HMGA2 and TGF-β/SMAD3 signaling pathways; knockdown of miR-26b expression inhibited VSMC proliferation in a SMAD3-dependent way (Yang et al., 2020). Experiments on the response to vascular injury showed that the loss of SMAD3 in mice resulted in enhanced intima hyperplasia. In addition, studies have shown expression of the lncRNA CRNDE to be downregulated in AAA tissues and angiotensin II-stimulated VSMCs. CRNDE overexpression promoted VSMC proliferation and repressed apoptosis in AAA by upregulating SMAD3 expression *via* B-cell lymphoma 3 (Kobayashi et al., 2005; Zhou et al., 2019). TGF-β/SMAD3 also interacts with canonical wingless type signaling to regulate the proliferation and apoptosis of SMCs (DiRenzo et al., 2016).

SMAD3 is also involved in regulation of some proinflammatory and cytokines. Connective-tissue growth factor (CTGF) is a key factor regulating ECM production. Peroxisome proliferator-activated receptor gamma binds directly to SMAD3 to inhibit induction of CTGF expression by TGF-β signaling (Fu et al., 2001). TGF-β1-based inhibition of iNOS and IL-6 expression was shown to be blocked completely in Smad3-deficient VSMCs (Feinberg et al., 2004). Those studies emphasized that the role of SMAD3 in SMCs is very complex. Indeed, the function of SMAD3 in lineage-specific VSMCs may be different. SMAD3 is essential for the differentiation of cardiovascular progenitor cell–VSMCs but not for the differentiation of neural crest stem cell–VSMCs. SMAD3 deficiency leads to reduced contractility of cardiovascular progenitor cell–VSMCs (Gong et al., 2020). It has also been reported that SMAD3 knockout resulted in myocardial-cell necrosis in mice with a 129 genetic background and aortic rupture in C57BL/6J mice: different genetic backgrounds may be responsible for these differences (Kashyap et al., 2017).

### Co-SMAD

#### -SMAD4 Gene

As a key transcription factor of the TGF-β signaling pathway, SMAD4 is essential for developmental and postnatal cardiovascular homeostasis (Lan et al., 2007; Xu et al., 2019). SMAD4 loss-of-function mutations are associated with juvenile polyposis syndrome (JPS) and a combined JPS-hereditary hemorrhagic telangiectasia (HHT) known as “JPS-HHT syndrome”, and some affected individuals present with aortic dilatation (Larsen Haidle et al., 1993; Jelsig et al., 2016). Several genetic studies have shown that Smad4 mutation is a risk factor for aortic dilatation in JPS-HHT (Andrabi et al., 2011; Teekakirikul et al., 2013; Wu, 2017; Inoguchi et al., 2019).

A single nucleotide polymorphism (SNP) rs12455792 located in the transcription-factor binding site of SMAD4 leads to decreased transcription activity and proteoglycan degradation, apoptosis of vascular smooth muscle cells (VSMCs) and fiber accumulation, which are involved in the pathological progression of TAAD (Wang et al., 2018). SMAD4 missense variants c.290G > T, p. (Arg97Leu) have been identified in one family associated with thoracic aortic disease. Experiments based on adenovirus transfection have revealed that the mutated site leads to increased ubiquitination-mediated degradation of Smad4 protein in SMCs (Duan et al., 2019). However, patients do not show the typical characteristics of JPS or HHT, though few cases have been reported (Schepers et al., 2018). These observations suggest that SMAD4 mutations are associated independently with aortic dilation. In a clinical analysis, comparing the degree of dilation of aortic roots and the ascending aorta between other HHT patients (ENG and ACVRL1 mutations) and non-HHT controls, SMAD4 mutation was found to be a significant risk factor (Vorselaars et al., 2017). Those studies demonstrated that SMAD4 mutations predispose to aortic dilatation, which suggests that screening for cardiovascular disease is necessary for JPS-HHT patients to prevent premature death due to AD or aortic rupture, which is clinically important. However, gain-of-function mutations in SMAD4 lead to Myhre syndrome; a rare, distinctive syndrome that mostly affects patients with cardiovascular abnormalities who do not have aortic dilatation (Lin et al., 2016; Meerschaut et al., 2019). The evidence stated above further emphasizes the importance of SMAD4 in the cardiovascular system.

With regard to the importance of SMAD4 and SMCs in the aorta, we conducted a mouse experiment to study how SMAD4 participates in AAs and ADs. SMAD4 deletion in SMCs led to AAs and ADs, which suggested a direct correlation between SMAD4 and AAs and ADs. Immunohistochemical assays showed that, in Smad4-deficient mice, many macrophages and cluster of differentiation (CD)4^+^T cells invaded the aorta, and expression of SMC-derived proinflammatory factors and chemokines was increased significantly. Selective clearance of macrophages and blockade of a chemokine signaling axis (CCL2-CCR2) could alleviate AAs in Smad4-deficient mice (Zhang et al., 2016). Those results suggest that the inflammatory response caused by SMAD4 loss in SMCs may be crucial for the pathogenesis of AAs and ADs. This view was confirmed by a subsequent study. Deletion of SMC-specific SMAD4 induced by tamoxifen in adult mice caused AA formation. SMAD4 ablation in SMCs activated inflammation early in the media and induced interleukin (IL)-1b production, and therapeutic targeting of IL-1b improved aortic disease and AA dilation induced by SMC-specific SMAD4 inactivation (Da Ros et al., 2017). Interestingly, specific knockout of TGFBR2 in SMCs did not cause severe inflammatory responses and AA progression was much more moderate in mice (Zhang et al., 2016). Those data suggested that compensatory activation of TGF-β non-canonical signaling in Smad4-deficient mice contributed significantly to the “inflammatory outburst” in the aorta.

MicroRNAs also play an important role in cardiovascular disease (Tang et al., 2021). Several researchers have studied the involvement of microRNAs (miRs) or long noncoding RNAs (lncRNAs) targeting SMAD4 in AAs and ADs. miR-146a-5p expression in circulating blood was increased significantly in patients with thoracic aortic dissection, and upregulated expression of miR-146a-5p targeted SMAD4 to promote the proliferation and migration of VSMCs (Xue et al., 2019). The lncRNA SNHG5 acts as a “molecular sponge” for miR-205, and its expression is downregulated in abdominal aortic aneurysm (AAA) patients. Downregulation of SNHG5 expression enhances the inhibitory effect of miR-205–-5p on SMAD4 and, thus, inhibits the proliferation, migration, and apoptosis of VSMCs (Nie et al., 2021). miR-26a expression has also been found to be downregulated in two mouse models of AAA and human aortic SMCs. Inhibition of miR-26A expression can increase SMAD4 expression, which enhances switching from a contractile phenotype to a synthetic phenotype (Leeper et al., 2011).

### I- SMAD Genes

#### -SMAD6 Gene

As an inhibitory SMAD, SMAD6 is involved in regulation of the TGF-β signaling pathway through various pathways. SMAD6 inhibits the formation of heteromerization with SMAD4 by binding to phosphorylated-SMAD2, but does not inhibit the activity of SMAD3. It also negatively regulates non-canonical TGF-β1 signaling by recruiting the deubiquitinase A20 to TRAF6 and inhibiting the TAK1-p38 MAPK/JNK pathway (Hata et al., 1998; Jung et al., 2013). Studies have revealed a crucial role for SMAD6 in the development and homeostasis of the cardiovascular system in Smad6-mutant mice. SMAD6 mutants have hyperplasia of cardiac valves and septation defects of outflow tracts, which is indicated by the development of aortic ossification and increased blood pressure (Galvin et al., 2000; Wylie et al., 2018). Individuals with a SMAD6 missense mutation present a complex cardiac pathological phenotype, such as aortic isthmus stenosis, a severe cardiac phenotype, and dilated cardiomyopathy (Kloth et al., 2019). SMAD6 is closely related to the occurrence of a bicuspid aortic valve (BAV). The latter is a heterogeneous disorder inherited primarily in an autosomal-dominant pattern with incomplete penetrance and variable expressivity. TAAs or acute ADs are common complications in inpatients with BAV. NOTCH1, TGFBR2, ACTA2, GATA5, NKX2.5, SMAD6, or ROBO4 mutations are the most commonly identified genetic causes of familial non-syndromic BAV (Musfee et al., 2020). However, there is little direct evidence that SMAD6 mutations in individuals lead to AAs or ADs. Whole-exome sequencing in 129 cases of sporadic TAAD revealed a 58-year-old male with a SMAD6 mutation (Li et al., 2021a). A clinical analysis showed limited contribution of the SMAD6 mutation to TAA development in BAV patients (Luyckx et al., 2019). Those data suggest that there may be other sites of genetic susceptibility involved in TAA. How SMAD6 participates in AAs requires *in vitro* and *in vivo* evidence.

#### -SMAD7 Gene

SMAD7 is a key negative regulator of TGF-β signaling, it antagonizes TGF-β signaling through multiple mechanisms in the context of different cell types, and altered expression of SMAD7 is often associated with cancer, tissue fibrosis and inflammatory diseases (Miyazawa and Miyazono, 2017). However, the genetic association between SMAD7 and aortic aneurysm and dissection has not been identified to date. Some studies suggest that SMAD7 also plays an important role in the cardiovascular system. Deletion of the MH2 domain of SMAD7 produced embryos with outflow tract, ventricular septum, ventricular compaction, and cardiac function defects (Chen et al., 2009).

Overexpression of SMAD7 suppressed differentiation and proliferation of VSMCs and reiterated defects in adult BMP9/10 double mutants mice (Wang et al., 2021). Homozygous knockdown of SMAD7 produced primarily fourth-related arch artery defects (Papangeli and Scambler, 2013), which indicates that SMAD7 is required for great vessel development. In addition, SMAD7 can be a microRNA target involved in SMCs function maintenance. Lentivirus knockdown of SMAD7 prevented AngII-induced Smad3^+/−^Mir-21^−/−^ mouse TAAD formation (Huang et al., 2018), and VSMC-specific miRNA-214 knockout inhibits AngII-induced hypertension through up-regulation of SMAD7 (Li et al., 2021b). The *in vivo* role of SMAD7 in the maintenance of aortic structure and function remains to be explored.

### Medical Therapy Targeting Abnormal TGF-β Signaling

Progressive expansion of AAs can lead to life-threatening aortic dissection and aortic rupture. So far, surgery or endovascular intervention has been the primary treatment, but medical therapy also plays an important role in slowing AAs dilation, and as the pathogenesis of AAs becomes clearer, it is expected that more effective drug therapy will be available.

Substantial evidence shows that the canonical TGF-β signaling abnormalities plays a central role in the formation of hereditary AAs, higher circulating TGF-β levels are associated with more advanced stages and higher rates of aortic dilatation in TAA patients (Franken et al., 2015). In addition, it interacts with the Renin-angiotensin system (RAS) at multiple levels. Ang II can directly increase TGF-β mRNA production, TGF-β protein expression and TGF-β activity upstream of signal. Abnormal upregulation of ACE and elevated local RAS signaling has been demonstrated in patients with AAs induced by abnormalities of the TGF-β classical signaling and in mouse models (van Dorst et al., 2021). Therefore, these two signaling pathways are important targets for drug therapy. Such as TGF-β neutralizing antibodies or Ang II type 1 receptor blocker (ARB) losartan. In addition, irbesartan, statins, and β-blockers are also common treatment options.

Almost all drug treatments have performed in MFS individuals, which provides a paradigm for LDS and other types of AAs, although the mutated gene loci are different, abnormal TGF-β signaling enhancement are a common feature. Several studies have shown that aortic dilatation was decreased after the use of neutralizing inhibitors against TGF-β or ARB in MFS mouse model (Yang et al., 2009; Habashi et al., 2011). Based on the validity of animal experiments, the first clinical trial evaluated the treatment effect of ARB on 18 pediatric patients with MFS, the use of ARB therapy significantly slowed the rate of progressive aortic-root dilation, but the distal ascending aorta is not affected. The results were confirmed by several subsequent randomized controlled clinical trials, and the beneficial effects of losartan are independent of its effects on blood pressure (Brooke et al., 2008; Groenink et al., 2013; Lacro et al., 2014). It is speculated that the inhibitory effect on TGF-β and ERK1/2-signaling may be the mechanism of losartan slowing down the progression of aortic aneurysms.

Several clinical trials have compared the efficacy of ARBs versus β-blockers in preventing aortic dilation in patients with MFS. Losartan was compared with the β-blocker atenolol in 608 patients with MFS, demonstrating that both drugs were equally effective in reducing aortic root dilation. Overall, the combined use of ARBs and β-Blockers not only significantly delayed aortic aneurysm dilation, but also prevented long-term adverse outcomes, according to a large number of clinical trials (Lacro et al., 2014; Forteza et al., 2016). Unfortunately, the clinical therapeutic effect of ARBs on MFS patients is not as significant as that of preclinical results. The time window of treatment, the type and dose of inhibitors used, and genetic heterogeneity in MFS patients may be responsible for this difference. Therefore, the effect of ARBs on genetic AA patients other than MFS needs to be carefully evaluated in consideration of the difference in the impact of gene heterogeneity on the therapeutic effect. It is necessary to conduct more genetic tests and studies on these individual patients.

## Discussion and Perspectives

TGF-β signaling pathway plays a central role in aortic diseases, but the mechanism is very complex. The genes TGFBR1, TGFBR2, SMAD2, SMAD3, SMAD4, SMAD6 and SMAD7 reviewed in this paper. Although they belong to the same TGF-β canonical signaling core genes family, patients with different gene mutations present very different clinical phenotype. A common phenotype of these gene mutations is aortic aneurysm or dissection, and aneurysm rupture is the main cause of death in these mutation patients. Therefore, in the clinical examination, it is necessary to screen for the cardiovascular system, especially the aorta, in patients with these mutations.

Loss-of-function mutations or deficiency of SMAD2, SMAD3, and SMAD4 genes in mice or humans lead to the formation of aortic aneurysms or dissections, suggesting that basal TGF-βsignaling canonical signaling is indispensable for maintaining the integrity of aortic structure and function. Functional abnormalities of VSMCs, a core component of the aortic structure, caused by SMADs mutations are the most well studied, these SMADs mutations cause VSMCs to switch from contractile to secretory. Another common mechanism is activation of non-canonical signaling pathways, such as ERK1/2 and MAPK, which initiate severe inflammatory responses and protease secretion, leading to aortic ECM remodeling. Interestingly, although only a few clinical cases have been reported and the permeability is low, the mutation of I-Smad SMAD6 still leads to the formation of aortic aneurysms. This further increases the complexity of the role of TGF-βsignaling pathway in maintaining aortic structure. Whether loss of SMAD6 leads to overactivation of non-canonical signaling pathways? As far as we know, there is no AAs mouse model caused by SMAD6 gene deletion at present, and its mechanism needs to be further explored in the future.

On the other hand, although the aortic tissues of these mutated patients showed abnormal enhancement of TGF-β signaling pathways, studies of molecular mechanisms in mouse models and cells suggest that the pathogenesis is very different.

Current drug therapies may only be suitable for a small subset of high-risk patients, and a better understanding of the underlying genetic mechanisms will help identify patient populations that respond to specific treatments. This heterogeneity of pathogenesis with different mutated genes means that targeted therapies may be the trend of the future. More efficient gene sequencing and editing techniques make this trend possible. In the study of mechanism, the efficient construction of locus mutation mouse model can better recapitulate the real human disease situation, which is more convenient for the study of pathogenesis and evaluation of potential drug targets, which will contribute to the development of personalized drug therapy.

## References

[B1] Andrabi S. Bekheirnia M. R. Robbins-Furman P. Lewis R. A. Prior T. W. Potocki L. (2011). SMAD4 Mutation Segregating in a Family with Juvenile Polyposis, Aortopathy, and Mitral Valve Dysfunction. Am. J. Med. Genet. A. 155A (5), 1165–1169. 10.1002/ajmg.a.33968 21465659

[B2] Angelov S. N. Hu J. H. Wei H. Airhart N. Shi M. Dichek D. A. (2017). TGF-β (Transforming Growth Factor-β) Signaling Protects the Thoracic and Abdominal Aorta from Angiotensin II-Induced Pathology by Distinct Mechanisms. Atvb 37 (11), 2102–2113. 10.1161/ATVBAHA.117.309401 PMC5658248 28729364

[B3] Bossone E. Eagle K. A. (2021). Epidemiology and Management of Aortic Disease: Aortic Aneurysms and Acute Aortic Syndromes. Nat. Rev. Cardiol. 18 (5), 331–348. 10.1038/s41569-020-00472-6 33353985

[B4] Brooke B. S. Habashi J. P. Judge D. P. Patel N. Loeys B. Dietz H. C. 3rd (2008). Angiotensin II Blockade and Aortic-Root Dilation in Marfan′s Syndrome. N. Engl. J. Med. 358 (26), 2787–2795. 10.1056/NEJMoa0706585 18579813 PMC2692965

[B5] Cannaerts E. Kempers M. Maugeri A. Marcelis C. Gardeitchik T. Richer J. (2019). Novel Pathogenic SMAD2 Variants in Five Families with Arterial Aneurysm and Dissection: Further Delineation of the Phenotype. J. Med. Genet. 56 (4), 220–227. 10.1136/jmedgenet-2018-105304 29967133

[B6] Chang H. Brown C. W. Matzuk M. M. (2002). Genetic Analysis of the Mammalian Transforming Growth Factor-Beta Superfamily. Endocr. Rev. 23 (6), 787–823. 10.1210/er.2002-0003 12466190

[B7] Chen Q. Chen H. Zheng D. Kuang C. Fang H. Zou B. (2009). Smad7 Is Required for the Development and Function of the Heart. J. Biol. Chem. 284 (1), 292–300. 10.1074/jbc.M807233200 18952608 PMC2610499

[B8] Da Ros F. Carnevale R. Cifelli G. Bizzotto D. Casaburo M. Perrotta M. (2017). Targeting Interleukin-1β Protects from Aortic Aneurysms Induced by Disrupted Transforming Growth Factor β Signaling. Immunity 47 (5), 959–e9. 10.1016/j.immuni.2017.10.016 29150241

[B9] Dai X. Shen J. Annam N. P. Jiang H. Levi E. Schworer C. M. (2015). SMAD3 Deficiency Promotes Vessel wall Remodeling, Collagen Fiber Reorganization and Leukocyte Infiltration in an Inflammatory Abdominal Aortic Aneurysm Mouse Model. Sci. Rep. 5, 10180. 10.1038/srep10180 25985281 PMC4434993

[B10] Derynck R. Zhang Y. E. (2003). Smad-dependent and Smad-independent Pathways in TGF-Beta Family Signalling. Nature 425 (6958), 577–584. 10.1038/nature02006 14534577

[B11] DiRenzo D. M. Chaudhary M. A. Shi X. Franco S. R. Zent J. Wang K. (2016). A Crosstalk between TGF-β/Smad3 and Wnt/β-Catenin Pathways Promotes Vascular Smooth Muscle Cell Proliferation. Cell Signal 28 (5), 498–505. 10.1016/j.cellsig.2016.02.011 26912210 PMC4788971

[B12] Duan X. Y. Guo D. C. Regalado E. S. Shen H. University of Washington Center for Mendelian G. Coselli J. S. (2019). SMAD4 Rare Variants in Individuals and Families with Thoracic Aortic Aneurysms and Dissections. Eur. J. Hum. Genet. 27 (7), 1054–1060. 10.1038/s41431-019-0357-x 30809044 PMC6777456

[B13] Feinberg M. W. Watanabe M. Lebedeva M. A. Depina A. S. Hanai J. Mammoto T. (2004). Transforming Growth Factor-Beta1 Inhibition of Vascular Smooth Muscle Cell Activation Is Mediated via Smad3. J. Biol. Chem. 279 (16), 16388–16393. 10.1074/jbc.M309664200 14754879

[B14] Feng X. H. Derynck R. (2005). Specificity and Versatility in Tgf-Beta Signaling through Smads. Annu. Rev. Cel Dev Biol 21, 659–693. 10.1146/annurev.cellbio.21.022404.142018 16212511

[B15] Forteza A. Evangelista A. Sánchez V. Teixidó-Turà G. Sanz P. Gutiérrez L. (2016). Efficacy of Losartan vs. Atenolol for the Prevention of Aortic Dilation in Marfan Syndrome: a Randomized Clinical Trial. Eur. Heart J. 37 (12), 978–985. 10.1093/eurheartj/ehv575 26518245

[B16] Franken R. Radonic T. den Hartog A. W. Groenink M. Pals G. van Eijk M. (2015). The Revised Role of TGF-β in Aortic Aneurysms in Marfan Syndrome. Neth. Heart J. 23 (2), 116–121. 10.1007/s12471-014-0622-0 25342281 PMC4315797

[B17] Fu M. Zhang J. Zhu X. Myles D. E. Willson T. M. Liu X. (2001). Peroxisome Proliferator-Activated Receptor Gamma Inhibits Transforming Growth Factor Beta-Induced Connective Tissue Growth Factor Expression in Human Aortic Smooth Muscle Cells by Interfering with Smad3. J. Biol. Chem. 276 (49), 45888–45894. 10.1074/jbc.M105490200 11590167

[B18] Fujiwara T. Takeda N. Ishii S. Morita H. Komuro I. (2019). Unique Mechanism by Which TGFBR1 Variants Cause 2 Distinct System Diseases - Loeys-Dietz Syndrome and Multiple Self-Healing Squamous Epithelioma. Circ. Rep. 1 (11), 487–492. 10.1253/circrep.CR-19-0098 33693090 PMC7897567

[B19] Fukuda H. Aoki H. Yoshida S. Tobinaga S. Otsuka H. Shojima T. (2018). Characterization of SMAD2 Activation in Human Thoracic Aortic Aneurysm. Ann. Vasc. Dis. 11 (1), 112–119. 10.3400/avd.oa.17-00114 29682117 PMC5882351

[B20] Gallo E. M. Loch D. C. Habashi J. P. Calderon J. F. Chen Y. Bedja D. (2014). Angiotensin II-dependent TGF-β Signaling Contributes to Loeys-Dietz Syndrome Vascular Pathogenesis. J. Clin. Invest. 124 (1), 448–460. 10.1172/JCI69666 24355923 PMC3871227

[B21] Galvin K. M. Donovan M. J. Lynch C. A. Meyer R. I. Paul R. J. Lorenz J. N. (2000). A Role for Smad6 in Development and Homeostasis of the Cardiovascular System. Nat. Genet. 24 (2), 171–174. 10.1038/72835 10655064

[B22] Gomez D. Al Haj Zen A. Borges L. F. Philippe M. Gutierrez P. S. Jondeau G. (2009). Syndromic and Non-syndromic Aneurysms of the Human Ascending Aorta Share Activation of the Smad2 Pathway. J. Pathol. 218 (1), 131–142. 10.1002/path.2516 19224541

[B23] Gong J. Zhou D. Jiang L. Qiu P. Milewicz D. M. Chen Y. E. (2020). *In Vitro* Lineage-Specific Differentiation of Vascular Smooth Muscle Cells in Response to SMAD3 Deficiency: Implications for SMAD3-Related Thoracic Aortic Aneurysm. Arterioscler Thromb. Vasc. Biol. 40 (7), 1651–1663. 10.1161/ATVBAHA.120.313033 32404006 PMC7316596

[B24] Goto K. Kamiya Y. Imamura T. Miyazono K. Miyazawa K. (2007). Selective Inhibitory Effects of Smad6 on Bone Morphogenetic Protein Type I Receptors. J. Biol. Chem. 282 (28), 20603–20611. 10.1074/jbc.M702100200 17493940

[B25] Goudie D. R. D'Alessandro M. Merriman B. Lee H. Szeverényi I. Avery S. (2011). Multiple Self-Healing Squamous Epithelioma Is Caused by a Disease-specific Spectrum of Mutations in TGFBR1. Nat. Genet. 43 (4), 365–369. 10.1038/ng.780 21358634

[B26] Goumans M. J. Valdimarsdottir G. Itoh S. Lebrin F. Larsson J. Mummery C. (2003). Activin Receptor-like Kinase (ALK)1 Is an Antagonistic Mediator of Lateral TGFbeta/ALK5 Signaling. Mol. Cel 12 (4), 817–828. 10.1016/s1097-2765(03)00386-1 14580334

[B27] Granadillo J. L. Chung W. K. Hecht L. Corsten-Janssen N. Wegner D. Nij Bijvank S. W. A. (2018). Variable Cardiovascular Phenotypes Associated with SMAD2 Pathogenic Variants. Hum. Mutat. 39 (12), 1875–1884. 10.1002/humu.23627 30157302

[B28] Groenink M. den Hartog A. W. Franken R. Radonic T. de Waard V. Timmermans J. (2013). Losartan Reduces Aortic Dilatation Rate in Adults with Marfan Syndrome: a Randomized Controlled Trial. Eur. Heart J. 34 (45), 3491–3500. 10.1093/eurheartj/eht334 23999449

[B29] Habashi J. P. Doyle J. J. Holm T. M. Aziz H. Schoenhoff F. Bedja D. (2011). Angiotensin II Type 2 Receptor Signaling Attenuates Aortic Aneurysm in Mice through ERK Antagonism. Science 332 (6027), 361–365. 10.1126/science.1192152 21493863 PMC3097422

[B30] Hanyu A. Ishidou Y. Ebisawa T. Shimanuki T. Imamura T. Miyazono K. (2001). The N Domain of Smad7 Is Essential for Specific Inhibition of Transforming Growth Factor-Beta Signaling. J. Cel Biol 155 (6), 1017–1027. 10.1083/jcb.200106023 PMC2150897 11739411

[B31] Hata A. Chen Y. G. (2016). TGF-β Signaling from Receptors to Smads. Cold Spring Harb Perspect. Biol. 8 (9). 10.1101/cshperspect.a022061 PMC5008074 27449815

[B32] Hata A. Lagna G. Massagué J. Hemmati-Brivanlou A. (1998). Smad6 Inhibits BMP/Smad1 Signaling by Specifically Competing with the Smad4 Tumor Suppressor. Genes Dev. 12 (2), 186–197. 10.1101/gad.12.2.186 9436979 PMC316444

[B33] Heldin C. H. Moustakas A. (2016). Signaling Receptors for TGF-β Family Members. Cold Spring Harb Perspect. Biol. 8 (8). 10.1101/cshperspect.a022053 PMC4968163 27481709

[B34] Horbelt D. Guo G. Robinson P. N. Knaus P. (2010). Quantitative Analysis of TGFBR2 Mutations in Marfan-Syndrome-Related Disorders Suggests a Correlation between Phenotypic Severity and Smad Signaling Activity. J. Cel Sci 123 (Pt 24), 4340–4350. 10.1242/jcs.074773 21098638

[B35] Hu J. H. Wei H. Jaffe M. Airhart N. Du L. Angelov S. N. (2015). Postnatal Deletion of the Type II Transforming Growth Factor-β Receptor in Smooth Muscle Cells Causes Severe Aortopathy in Mice. Arterioscler Thromb. Vasc. Biol. 35 (12), 2647–2656. 10.1161/ATVBAHA.115.306573 26494233 PMC4743752

[B36] Huang X. Yue Z. Wu J. Chen J. Wang S. Wu J. (2018). MicroRNA-21 Knockout Exacerbates Angiotensin II-Induced Thoracic Aortic Aneurysm and Dissection in Mice with Abnormal Transforming Growth Factor-β-SMAD3 Signaling. Arterioscler Thromb. Vasc. Biol. 38 (5), 1086–1101. 10.1161/ATVBAHA.117.310694 29519942

[B37] Inoguchi Y. Kaku B. Kitagawa N. Katsuda S. (2019). Hereditary Hemorrhagic Telangiectasia with SMAD4 Mutations Is Associated with Fatty Degeneration of the Left Ventricle, Coronary Artery Aneurysm, and Abdominal Aortic Aneurysm. Intern. Med. 58 (3), 387–393. 10.2169/internalmedicine.1287-18 30210120 PMC6395134

[B38] Isogai Z. Ono R. N. Ushiro S. Keene D. R. Chen Y. Mazzieri R. (2003). Latent Transforming Growth Factor Beta-Binding Protein 1 Interacts with Fibrillin and Is a Microfibril-Associated Protein. J. Biol. Chem. 278 (4), 2750–2757. 10.1074/jbc.M209256200 12429738

[B39] Isselbacher E. M. Lino Cardenas C. L. Lindsay M. E. (2016). Hereditary Influence in Thoracic Aortic Aneurysm and Dissection. Circulation 133 (24), 2516–2528. 10.1161/CIRCULATIONAHA.116.009762 27297344 PMC5031368

[B40] Jelsig A. M. Tørring P. M. Kjeldsen A. D. Qvist N. Bojesen A. Jensen U. B. (2016). JP-HHT Phenotype in Danish Patients with SMAD4 Mutations. Clin. Genet. 90 (1), 55–62. 10.1111/cge.12693 26572829

[B41] Jones J. A. Spinale F. G. Ikonomidis J. S. (2009). Transforming Growth Factor-Beta Signaling in Thoracic Aortic Aneurysm Development: a Paradox in Pathogenesis. J. Vasc. Res. 46 (2), 119–137. 10.1159/000151766 18765947 PMC2645475

[B42] Jung S. M. Lee J. H. Park J. Oh Y. S. Lee S. K. Park J. S. (2013). Smad6 Inhibits Non-canonical TGF-Β1 Signalling by Recruiting the Deubiquitinase A20 to TRAF6. Nat. Commun. 4, 2562. 10.1038/ncomms3562 24096742

[B43] Kashyap S. Warner G. Hu Z. Gao F. Osman M. Al Saiegh Y. (2017). Cardiovascular Phenotype in Smad3 Deficient Mice with Renovascular Hypertension. PLoS One 12 (10), e0187062. 10.1371/journal.pone.0187062 29073282 PMC5658153

[B44] Kloth K. Bierhals T. Johannsen J. Harms F. L. Juusola J. Johnson M. C. (2019). Biallelic Variants in SMAD6 Are Associated with a Complex Cardiovascular Phenotype. Hum. Genet. 138 (6), 625–634. 10.1007/s00439-019-02011-x 30963242

[B45] Kobayashi K. Yokote K. Fujimoto M. Yamashita K. Sakamoto A. Kitahara M. (2005). Targeted Disruption of TGF-Beta-Smad3 Signaling Leads to Enhanced Neointimal Hyperplasia with Diminished Matrix Deposition in Response to Vascular Injury. Circ. Res. 96 (8), 904–912. 10.1161/01.RES.0000163980.55495.44 15790953

[B46] Lacro R. V. Dietz H. C. Sleeper L. A. Yetman A. T. Bradley T. J. Colan S. D. (2014). Atenolol versus Losartan in Children and Young Adults with Marfan′s Syndrome. N. Engl. J. Med. 371 (22), 2061–2071. 10.1056/NEJMoa1404731 25405392 PMC4386623

[B47] Lan Y. Liu B. Yao H. Li F. Weng T. Yang G. (2007). Essential Role of Endothelial Smad4 in Vascular Remodeling and Integrity. Mol. Cel Biol 27 (21), 7683–7692. 10.1128/MCB.00577-07 PMC2169040 17724086

[B48] Larsen Haidle J. MacFarland S. P. Howe J. R. Adam M. P. Ardinger H. H. Pagon R. A. (1993). in Juvenile Polyposis SyndromeGeneReviews((R)). Seattle (WA).

[B49] Lee M. K. Pardoux C. Hall M. C. Lee P. S. Warburton D. Qing J. (2007). TGF-beta Activates Erk MAP Kinase Signalling through Direct Phosphorylation of ShcA. EMBO J. 26 (17), 3957–3967. 10.1038/sj.emboj.7601818 17673906 PMC1994119

[B50] Leeper N. J. Raiesdana A. Kojima Y. Chun H. J. Azuma J. Maegdefessel L. (2011). MicroRNA-26a Is a Novel Regulator of Vascular Smooth Muscle Cell Function. J. Cel Physiol 226 (4), 1035–1043. 10.1002/jcp.22422 PMC3108574 20857419

[B51] Li J. Li P. Zhang Y. Li G. B. Zhou Y. G. Yang K. (2013). c-Ski Inhibits the Proliferation of Vascular Smooth Muscle Cells via Suppressing Smad3 Signaling but Stimulating P38 Pathway. Cel Signal 25 (1), 159–167. 10.1016/j.cellsig.2012.09.001 22986000

[B52] Li W. Li Q. Jiao Y. Qin L. Ali R. Zhou J. (2014). Tgfbr2 Disruption in Postnatal Smooth Muscle Impairs Aortic wall Homeostasis. J. Clin. Invest. 124 (2), 755–767. 10.1172/JCI69942 24401272 PMC3904608

[B53] Li Y. Fang M. Yang J. Yu C. Kuang J. Sun T. (2021a). Analysis of the Contribution of 129 Candidate Genes to Thoracic Aortic Aneurysm or Dissection of a Mixed Cohort of Sporadic and Familial Cases in South China. Am. J. Transl Res. 13 (5), 4281–4295. 34150014 PMC8205813

[B54] Li Y. Li H. Xing W. Li J. Du R. Cao D. (2021b). Vascular Smooth Muscle Cell‐specific miRNA‐214 Knockout Inhibits Angiotensin II‐induced Hypertension through Upregulation of Smad7. FASEB j. 35 (11), e21947. 10.1096/fj.202100766RR 34637552

[B55] Li Z. Kong W. (2020). Cellular Signaling in Abdominal Aortic Aneurysm. Cel Signal 70, 109575. 10.1016/j.cellsig.2020.109575 32088371

[B56] Liao M. F. Gong Q. W. Liu L. Xiong X. Y. Zhang Q. Gong C. X. (2018). Association Between Polymorphism of SMAD3 Gene and Risk of Sporadic Intracranial Arterial Aneurysms in the Chinese Han Population. J. Clin. Neurosci. 47, 269–272. 10.1016/j.jocn.2017.09.006 28988651

[B57] Lin A. E. Michot C. Cormier-Daire V. L'Ecuyer T. J. Matherne G. P. Barnes B. H. (2016). Gain-of-Function Mutations in SMAD4 Cause a Distinctive Repertoire of Cardiovascular Phenotypes in Patients with Myhre Syndrome. Am. J. Med. Genet. A. 170 (10), 2617–2631. 10.1002/ajmg.a.37739 27302097

[B58] Lin F. Yang X. (2010). TGF-β Signaling in Aortic Aneurysm: Another Round of Controversy. J. Genet. Genomics 37 (9), 583–591. 10.1016/S1673-8527(09)60078-3 20933212

[B59] Lindsay M. E. Dietz H. C. (2014). The Genetic Basis of Aortic Aneurysm. Cold Spring Harb Perspect. Med. 4 (9), a015909. 10.1101/cshperspect.a015909 25183854 PMC4143103

[B60] Loeys B. L. Chen J. Neptune E. R. Judge D. P. Podowski M. Holm T. (2005). A Syndrome of Altered Cardiovascular, Craniofacial, Neurocognitive and Skeletal Development Caused by Mutations in TGFBR1 or TGFBR2. Nat. Genet. 37 (3), 275–281. 10.1038/ng1511 15731757

[B61] Loeys B. L. Schwarze U. Holm T. Callewaert B. L. Thomas G. H. Pannu H. (2006). Aneurysm Syndromes Caused by Mutations in the TGF-Beta Receptor. N. Engl. J. Med. 355 (8), 788–798. 10.1056/NEJMoa055695 16928994

[B62] Loinard C. Basatemur G. Masters L. Baker L. Harrison J. Figg N. (2014). Deletion of Chromosome 9p21 Noncoding Cardiovascular Risk Interval in Mice Alters Smad2 Signaling and Promotes Vascular Aneurysm. Circ. Cardiovasc. Genet. 7 (6), 799–805. 10.1161/CIRCGENETICS.114.000696 25176937

[B63] Luyckx I. MacCarrick G. Kempers M. Meester J. Geryl C. Rombouts O. (2019). Confirmation of the Role of Pathogenic SMAD6 Variants in Bicuspid Aortic Valve-Related Aortopathy. Eur. J. Hum. Genet. 27 (7), 1044–1053. 10.1038/s41431-019-0363-z 30796334 PMC6777625

[B64] MacCarrick G. Black J. H. 3rd Bowdin S. El-Hamamsy I. Frischmeyer-Guerrerio P. A. Guerrerio A. L. (2014). Loeys-Dietz Syndrome: a Primer for Diagnosis and Management. Genet. Med. 16 (8), 576–587. 10.1038/gim.2014.11 24577266 PMC4131122

[B65] MacFarlane E. G. Haupt J. Dietz H. C. Shore E. M. (2017). TGF-β Family Signaling in Connective Tissue and Skeletal Diseases. Cold Spring Harb Perspect. Biol. 9 (11). 10.1101/cshperspect.a022269 PMC5666637 28246187

[B66] Meerschaut I. Beyens A. Steyaert W. De Rycke R. Bonte K. De Backer T. (2019). Myhre Syndrome: A First Familial Recurrence and Broadening of the Phenotypic Spectrum. Am. J. Med. Genet. A. 179 (12), 2494–2499. 10.1002/ajmg.a.61377 31595668

[B67] Meester J. A. N. Verstraeten A. Schepers D. Alaerts M. Van Laer L. Loeys B. L. (2017). Differences in Manifestations of Marfan Syndrome, Ehlers-Danlos Syndrome, and Loeys-Dietz Syndrome. Ann. Cardiothorac. Surg. 6 (6), 582–594. 10.21037/acs.2017.11.03 29270370 PMC5721110

[B68] Meng X. M. Nikolic-Paterson D. J. Lan H. Y. (2016). TGF-β: the Master Regulator of Fibrosis. Nat. Rev. Nephrol. 12 (6), 325–338. 10.1038/nrneph.2016.48 27108839

[B69] Micha D. Guo D. C. Hilhorst-Hofstee Y. van Kooten F. Atmaja D. Overwater E. (2015). SMAD2 Mutations Are Associated with Arterial Aneurysms and Dissections. Hum. Mutat. 36 (12), 1145–1149. 10.1002/humu.22854 26247899

[B70] Miyazawa K. Miyazono K. (2017). Regulation of TGF-β Family Signaling by Inhibitory Smads. Cold Spring Harb Perspect. Biol. 9 (3). 10.1101/cshperspect.a022095 PMC5334261 27920040

[B71] Mizuguchi T. Collod-Beroud G. Akiyama T. Abifadel M. Harada N. Morisaki T. (2004). Heterozygous TGFBR2 Mutations in Marfan Syndrome. Nat. Genet. 36 (8), 855–860. 10.1038/ng1392 15235604 PMC2230615

[B72] Morikawa M. Derynck R. Miyazono K. (2016). TGF-β and the TGF-β Family: Context-dependent Roles in Cell and Tissue Physiology. Cold Spring Harb Perspect. Biol. 8 (5), a021873. 10.1101/cshperspect.a021873 27141051 PMC4852809

[B73] Moustakas A. Souchelnytskyi S. Heldin C. H. (2001). Smad Regulation in TGF-Beta Signal Transduction. J. Cel Sci 114 (Pt 24), 4359–4369. 10.1242/jcs.114.24.4359 11792802

[B74] Musfee F. I. Guo D. Pinard A. C. Hostetler E. M. Blue E. E. Nickerson D. A. (2020). Rare Deleterious Variants of NOTCH1, GATA4, SMAD6, and ROBO4 Are Enriched in BAV with Early Onset Complications but Not in BAV with Heritable Thoracic Aortic Disease. Mol. Genet. Genomic Med. 8 (10), e1406. 10.1002/mgg3.1406 32748548 PMC7549564

[B75] Nie H. Zhao W. Wang S. Zhou W. (2021). Based on Bioinformatics Analysis Lncrna SNHG5 Modulates the Function of Vascular Smooth Muscle Cells through mir-205-5p/SMAD4 in Abdominal Aortic Aneurysm. Immun. Inflamm. Dis. 9 (4), 1306–1320. 10.1002/iid3.478 34185955 PMC8589383

[B76] Pannu H. Fadulu V. T. Chang J. Lafont A. Hasham S. N. Sparks E. (2005). Mutations in Transforming Growth Factor-Beta Receptor Type II Cause Familial Thoracic Aortic Aneurysms and Dissections. Circulation 112 (4), 513–520. 10.1161/CIRCULATIONAHA.105.537340 16027248

[B77] Papangeli I. Scambler P. J. (2013). Tbx1 Genetically Interacts with the Transforming Growth Factor-Β/bone Morphogenetic Protein Inhibitor Smad7 during Great Vessel Remodeling. Circ. Res. 112 (1), 90–102. 10.1161/CIRCRESAHA.112.270223 23011393

[B78] Park S. H. (2005). Fine Tuning and Cross-Talking of TGF-Beta Signal by Inhibitory Smads. J. Biochem. Mol. Biol. 38 (1), 9–16. 10.5483/bmbrep.2005.38.1.009 15715940

[B79] Peng C. (2003). The TGF-Beta Superfamily and its Roles in the Human Ovary and Placenta. J. Obstet. Gynaecol. Can. 25 (10), 834–844. 10.1016/s1701-2163(16)30674-0 14532952

[B80] Perrella M. A. Jain M. K. Lee M. E. (1998). Role of TGF-Beta in Vascular Development and Vascular Reactivity. Miner Electrolyte Metab. 24 (2-3), 136–143. 10.1159/000057361 9525696

[B81] Qiu P. Ritchie R. P. Fu Z. Cao D. Cumming J. Miano J. M. (2005). Myocardin Enhances Smad3-Mediated Transforming Growth Factor-Beta1 Signaling in a CArG Box-independent Manner: Smad-Binding Element Is an Important Cis Element for SM22alpha Transcription *In Vivo* . Circ. Res. 97 (10), 983–991. 10.1161/01.RES.0000190604.90049.71 16224064

[B82] Rabkin S. W. (2017). The Role Matrix Metalloproteinases in the Production of Aortic Aneurysm. Prog. Mol. Biol. Transl Sci. 147, 239–265. 10.1016/bs.pmbts.2017.02.002 28413030

[B83] Regalado E. S. Guo D. C. Villamizar C. Avidan N. Gilchrist D. McGillivray B. (2011). Exome Sequencing Identifies SMAD3 Mutations as a Cause of Familial Thoracic Aortic Aneurysm and Dissection with Intracranial and Other Arterial Aneurysms. Circ. Res. 109 (6), 680–686. 10.1161/CIRCRESAHA.111.248161 21778426 PMC4115811

[B84] Sampson U. K. Norman P. E. Fowkes F. G. Aboyans V. Yanna Song S. Harrell F. E. Jr. (2014). Global and Regional burden of Aortic Dissection and Aneurysms: Mortality Trends in 21 World Regions, 1990 to 2010. Glob. Heart 9 (1), 171–e10. 10.1016/j.gheart.2013.12.010 25432126

[B85] Schepers D. Tortora G. Morisaki H. MacCarrick G. Lindsay M. Liang D. (2018). A Mutation Update on the LDS-Associated Genes TGFB2/3 and SMAD2/3. Hum. Mutat. 39 (5), 621–634. 10.1002/humu.23407 29392890 PMC5947146

[B86] Shi M. Zhu J. Wang R. Chen X. Mi L. Walz T. (2011). Latent TGF-β Structure and Activation. Nature 474 (7351), 343–349. 10.1038/nature10152 21677751 PMC4717672

[B87] Sorrentino A. Thakur N. Grimsby S. Marcusson A. von Bulow V. Schuster N. (2008). The Type I TGF-Beta Receptor Engages TRAF6 to Activate TAK1 in a Receptor Kinase-independent Manner. Nat. Cel Biol 10 (10), 1199–1207. 10.1038/ncb1780 18758450

[B88] Takeda N. Hara H. Fujiwara T. Kanaya T. Maemura S. Komuro I. (2018). TGF-β Signaling-Related Genes and Thoracic Aortic Aneurysms and Dissections. Int. J. Mol. Sci. 19 (7). 10.3390/ijms19072125 PMC6073540 30037098

[B89] Tan C. K. Tan E. H. Luo B. Huang C. L. Loo J. S. Choong C. (2013). SMAD3 Deficiency Promotes Inflammatory Aortic Aneurysms in Angiotensin II-Infused Mice via Activation of iNOS. J. Am. Heart Assoc. 2 (3), e000269. 10.1161/JAHA.113.000269 23782924 PMC3698794

[B90] Tang Y. Fan W. Zou B. Yan W. Hou Y. Kwabena Agyare O. (2021). TGF-β Signaling and microRNA Cross-Talk Regulates Abdominal Aortic Aneurysm Progression. Clin. Chim. Acta 515, 90–95. 10.1016/j.cca.2020.12.031 33388307

[B91] Teekakirikul P. Milewicz D. M. Miller D. T. Lacro R. V. Regalado E. S. Rosales A. M. (2013). Thoracic Aortic Disease in Two Patients with Juvenile Polyposis Syndrome and SMAD4 Mutations. Am. J. Med. Genet. A. 161A (1), 185–191. 10.1002/ajmg.a.35659 23239472 PMC3535513

[B92] Tran-Fadulu V. Pannu H. Kim D. H. Vick G. W. 3rd Lonsford C. M. Lafont A. L. (2009). Analysis of Multigenerational Families with Thoracic Aortic Aneurysms and Dissections Due to TGFBR1 or TGFBR2 Mutations. J. Med. Genet. 46 (9), 607–613. 10.1136/jmg.2008.062844 19542084

[B93] Tzavlaki K. Moustakas A. (2020). TGF-β Signaling. Biomolecules 10 (3). 10.3390/biom10030487 PMC7175140 32210029

[B94] van de Laar I. M. Oldenburg R. A. Pals G. Roos-Hesselink J. W. de Graaf B. M. Verhagen J. M. (2011). Mutations in SMAD3 Cause a Syndromic Form of Aortic Aneurysms and Dissections with Early-Onset Osteoarthritis. Nat. Genet. 43 (2), 121–126. 10.1038/ng.744 21217753

[B95] van de Laar I. M. van der Linde D. Oei E. H. Bos P. K. Bessems J. H. Bierma-Zeinstra S. M. (2012). Phenotypic Spectrum of the SMAD3-Related Aneurysms-Osteoarthritis Syndrome. J. Med. Genet. 49 (1), 47–57. 10.1136/jmedgenet-2011-100382 22167769

[B96] van der Linde D. van de Laar I. M. Bertoli-Avella A. M. Oldenburg R. A. Bekkers J. A. Mattace-Raso F. U. (2012). Aggressive Cardiovascular Phenotype of Aneurysms-Osteoarthritis Syndrome Caused by Pathogenic SMAD3 Variants. J. Am. Coll. Cardiol. 60 (5), 397–403. 10.1016/j.jacc.2011.12.052 22633655

[B97] van Dorst D. C. H. de Wagenaar N. P. van der Pluijm I. Roos-Hesselink J. W. Essers J. Danser A. H. J. (2021). Transforming Growth Factor-β and the Renin-Angiotensin System in Syndromic Thoracic Aortic Aneurysms: Implications for Treatment. Cardiovasc. Drugs Ther. 35 (6), 1233–1252. 10.1007/s10557-020-07116-4 33283255 PMC8578102

[B98] Van Laer L. Dietz H. Loeys B. (2014). Loeys-Dietz Syndrome. Adv. Exp. Med. Biol. 802, 95–105. 10.1007/978-94-007-7893-1_7 24443023

[B99] Vorselaars V. M. M. Diederik A. Prabhudesai V. Velthuis S. Vos J. A. Snijder R. J. (2017). SMAD4 Gene Mutation Increases the Risk of Aortic Dilation in Patients with Hereditary Haemorrhagic Telangiectasia. Int. J. Cardiol. 245, 114–118. 10.1016/j.ijcard.2017.06.059 28874282

[B100] Waldrip W. R. Bikoff E. K. Hoodless P. A. Wrana J. L. Robertson E. J. (1998). Smad2 Signaling in Extraembryonic Tissues Determines Anterior-Posterior Polarity of the Early Mouse Embryo. Cell 92 (6), 797–808. 10.1016/s0092-8674(00)81407-5 9529255

[B101] Wang L. Rice M. Swist S. Kubin T. Wu F. Wang S. (2021). BMP9 and BMP10 Act Directly on Vascular Smooth Muscle Cells for Generation and Maintenance of the Contractile State. Circulation 143 (14), 1394–1410. 10.1161/CIRCULATIONAHA.120.047375 33334130

[B102] Wang Y. Yin P. Chen Y. H. Yu Y. S. Ye W. X. Huang H. Y. (2018). A Functional Variant of SMAD4 Enhances Macrophage Recruitment and Inflammatory Response via TGF-β Signal Activation in Thoracic Aortic Aneurysm and Dissection. Aging (Albany NY) 10 (12), 3683–3701. 10.18632/aging.101662 30530919 PMC6326647

[B103] Weinstein M. Yang X. Li C. Xu X. Gotay J. Deng C. X. (1998). Failure of Egg cylinder Elongation and Mesoderm Induction in Mouse Embryos Lacking the Tumor Suppressor Smad2. Proc. Natl. Acad. Sci. U S A. 95 (16), 9378–9383. 10.1073/pnas.95.16.9378 9689088 PMC21346

[B104] Wilkes M. C. Mitchell H. Penheiter S. G. Doré J. J. Suzuki K. Edens M. (2005). Transforming Growth Factor-Beta Activation of Phosphatidylinositol 3-kinase Is Independent of Smad2 and Smad3 and Regulates Fibroblast Responses via P21-Activated Kinase-2. Cancer Res. 65 (22), 10431–10440. 10.1158/0008-5472.CAN-05-1522 16288034

[B105] Wrana J. L. Attisano L. Cárcamo J. Zentella A. Doody J. Laiho M. (1992). TGF Beta Signals through a Heteromeric Protein Kinase Receptor Complex. Cell 71 (6), 1003–1014. 10.1016/0092-8674(92)90395-s 1333888

[B106] Wu J. Niu J. Li X. Wang X. Guo Z. Zhang F. (2014). TGF-β1 Induces Senescence of Bone Marrow Mesenchymal Stem Cells via Increase of Mitochondrial ROS Production. BMC Dev. Biol. 14, 21. 10.1186/1471-213X-14-21 24886313 PMC4031602

[B107] Wu L. (2017). Functional Characteristics of a Novel SMAD4 Mutation from Thoracic Aortic Aneurysms (TAA). Gene 628, 129–133. 10.1016/j.gene.2017.07.042 28716708

[B108] Wylie L. A. Mouillesseaux K. P. Chong D. C. Bautch V. L. (2018). Developmental SMAD6 Loss Leads to Blood Vessel Hemorrhage and Disrupted Endothelial Cell Junctions. Dev. Biol. 442 (2), 199–209. 10.1016/j.ydbio.2018.07.027 30098998 PMC6903908

[B109] Xie W. B. Li Z. Shi N. Guo X. Tang J. Ju W. (2013). Smad2 and Myocardin-Related Transcription Factor B Cooperatively Regulate Vascular Smooth Muscle Differentiation from Neural Crest Cells. Circ. Res. 113 (8), e76–86. 10.1161/CIRCRESAHA.113.301921 23817199 PMC3837448

[B110] Xu J. Gruber P. J. Chien K. R. (2019). SMAD4 Is Essential for Human Cardiac Mesodermal Precursor Cell Formation. Stem Cells 37 (2), 216–225. 10.1002/stem.2943 30376214 PMC7379516

[B111] Xue L. Luo S. Ding H. Liu Y. Huang W. Fan X. (2019). Upregulation of miR-146a-5p Is Associated with Increased Proliferation and Migration of Vascular Smooth Muscle Cells in Aortic Dissection. J. Clin. Lab. Anal. 33 (4), e22843. 10.1002/jcla.22843 30779466 PMC6528573

[B112] Yamashita M. Fatyol K. Jin C. Wang X. Liu Z. Zhang Y. E. (2008). TRAF6 Mediates Smad-independent Activation of JNK and P38 by TGF-Beta. Mol. Cel 31 (6), 918–924. 10.1016/j.molcel.2008.09.002 PMC2621323 18922473

[B113] Yang H. H. Kim J. M. Chum E. van Breemen C. Chung A. W. (2009). Long-term Effects of Losartan on Structure and Function of the Thoracic Aorta in a Mouse Model of Marfan Syndrome. Br. J. Pharmacol. 158 (6), 1503–1512. 10.1111/j.1476-5381.2009.00443.x 19814725 PMC2795217

[B114] Yang P. Schmit B. M. Fu C. DeSart K. Oh S. P. Berceli S. A. (2016). Smooth Muscle Cell-specific Tgfbr1 Deficiency Promotes Aortic Aneurysm Formation by Stimulating Multiple Signaling Events. Sci. Rep. 6, 35444. 10.1038/srep35444 27739498 PMC5064316

[B115] Yang P. Wu P. Liu X. Feng J. Zheng S. Wang Y. (2020). MiR-26b Suppresses the Development of Stanford Type A Aortic Dissection by Regulating HMGA2 and TGF-β/Smad3 Signaling Pathway. Ann. Thorac. Cardiovasc. Surg. 26 (3), 140–150. 10.5761/atcs.oa.19-00184 31723084 PMC7303312

[B116] Ye P. Chen W. Wu J. Huang X. Li J. Wang S. (2013). GM-CSF Contributes to Aortic Aneurysms Resulting from SMAD3 Deficiency. J. Clin. Invest. 123 (5), 2317–2331. 10.1172/JCI67356 23585475 PMC3635740

[B117] Zhang P. Hou S. Chen J. Zhang J. Lin F. Ju R. (2016). Smad4 Deficiency in Smooth Muscle Cells Initiates the Formation of Aortic Aneurysm. Circ. Res. 118 (3), 388–399. 10.1161/CIRCRESAHA.115.308040 26699655

[B118] Zhang W. Zeng Q. Xu Y. Ying H. Zhou W. Cao Q. (2017). Exome Sequencing Identified a Novel SMAD2 Mutation in a Chinese Family with Early Onset Aortic Aneurysms. Clin. Chim. Acta 468, 211–214. 10.1016/j.cca.2017.03.007 28283438

[B119] Zhang W. Zhou M. Liu C. Liu C. Qiao T. Huang D. (2015). A Novel Mutation of SMAD3 Identified in a Chinese Family with Aneurysms-Osteoarthritis Syndrome. Biomed. Res. Int. 2015, 968135. 10.1155/2015/968135 26221609 PMC4499615

[B120] Zhou Y. He X. Liu R. Qin Y. Wang S. Yao X. (2019). LncRNA CRNDE Regulates the Proliferation and Migration of Vascular Smooth Muscle Cells. J. Cel Physiol 234, 16205–16214. 10.1002/jcp.28284 30740670

